# Comparison of the virtual reality and mobile games regarding physiological load

**DOI:** 10.3389/fphys.2025.1556434

**Published:** 2025-04-03

**Authors:** Bilal Biçer, Berkay Löklüoğlu

**Affiliations:** Faculty of Sport Sciences, Hatay Mustafa Kemal University, Antakya, Hatay, Türkiye

**Keywords:** heart rate, physiological load, virtual reality game, mobile game, physical activity

## Abstract

**Introduction:**

Physical inactivity or lack of physical activity has become an increasingly widespread and important global public health problem. Along with technological developments, the interest in e-sports is also increasing. Virtual reality (VR) games applied within the scope of e-sports include physical movements by nature, which encourages players to move more and be active. This study aimed to compare VR and mobile games regarding physiologic load.

**Methods:**

Twenty-two (13 males, 9 females) healthy university students with a mean age of 21.72 ± 1.39 years, height of 171.59 ± 9.12 cm, and body weight of 64.95 ± 13.35 kg, who did not smoke, did not play sports professionally, and did not have chronic diseases, voluntarily participated in the study. Participants were randomly assigned to play an active video game (VR) using a virtual reality headset and a mobile game (MO) on a smartphone for 2 days. The game duration was determined as 15 min. The body temperature of the participants before and after the game and heart rate (HR) values during the game were measured from beat to beat. A non-contact infrared thermometer was used for measuring body temperature, and a telemetric device was used for HR measurements.

**Results:**

When VR and MO games were compared regarding body temperature, no intra- and inter-group differences were observed. Regarding HR, HR_mean_, HR_max_, and HR_total_ values of the VR game were statistically significantly higher than MO games. In addition, in the analyses performed according to the time spent in the percentage of maximal heart rate, it was observed that the time spent in the MO game was significantly longer compared to the VR game below the Under Very Light (UVL). On the contrary, the time spent in the VR game was significantly longer than the MO game at Above Very Light (AVL).

**Conclusion:**

As a result, the heart rate parameters measured in the VR game were higher than in the MO game at all levels, suggesting that VR may be an effective tool for physical activity-based gaming experiences.

## 1 Introduction

Physical inactivity or lack of physical activity is a global health problem becoming increasingly prevalent in society (Moxley et al., 2022). However, research shows that regular physical activity is essential and beneficial for improving muscular and cardiorespiratory fitness, improving bone and functional health, and reducing the risk of many chronic diseases and some types of cancer. Therefore, physical activity is the cornerstone of a healthy lifestyle (Giroir and Wright, 2018; Chalkley and Milton, 2021). The importance of movement and physical activity for all age groups is known (Janssen and LeBlanc, 2010). However, with technological developments, the interest and addiction to playing games is increasing, especially in children and young people. Mobile (MO) games played with smartphones mainly cause limitations in movement and physical activity for this population. The proliferation of virtual reality (VR) and MO games with technological advances has generated significant interest in understanding their physiological effects. While both platforms offer immersive experiences, their technological capabilities and effects on users differ (Vatsal et al., 2024).

Virtual reality (VR) is a computer-generated simulation of a three-dimensional environment that can be interacted with seemingly real or physically using specialized electronic equipment such as a helmet with a screen in front, a pair of goggles, or gloves equipped with sensors (Luo et al., 2021). They can be used for sports training, rehabilitation applications, and entertainment. MO games are played on mobile devices such as smartphones and tablets. Unlike VR games, they do not require body movement and are played only with the movements of the fingers and wrists (Yamaguchi, 2023). In recent years, technological advances, increased accessibility, and mobility of VR systems have led to a great interest in VR applications in a wide range of recreational and high-performance areas, including sports, which is increasing (Richlan et al., 2023).

Studies examining the effects of VR and MO games in different aspects are available in the literature. Studies have been conducted on the effects of VR games on actual and perceived exertion (Stewart et al., 2022), movement skill and balance development (Neri et al., 2017), sprint and jump performance (Nambi et al., 2020), and heart rate (McClure and Schofield, 2019). Unlike MO games, VR games include various body movements and enable physical activity (Ramírez-Granizo et al., 2020). The impact of MO games on physiological parameters has been less extensively studied than VR, but some research suggests that long-term use can lead to adverse health effects. Pandey and Vaishnav (2023) conducted a systematic review of studies on the health effects of digital devices on children. They highlighted musculoskeletal problems, sleep disturbances, headaches, eye strain, impaired concentration, and memory loss as potential consequences of excessive mobile gaming (Pandey and Vaishnav, 2023). The physiological effects of MO and VR gaming differ in degree and type. One reason lies in the potential for physical activity in VR games. VR games may involve physical interactions, increasing heart rate and exertion (Tan et al., 2024), whereas MO games are typically less physically demanding (Caldas et al., 2020).

VR games inherently involve physical movement, encouraging players to move more and be active. These games often include full-body movements that increase cardiovascular activity, improve coordination, and engage multiple muscle groups. VR games can, therefore, be a powerful tool to promote physical activity (Giakoni-Ramírez et al., 2023). VR games, characterized by their immersive and interactive nature, have been shown to cause significant physiological changes compared to traditional screen-based games. Studies have consistently shown increased levels of arousal in VR game players. VR games have been reported to elicit significantly higher arousal, cognitive load, and stress with lower dominance compared to flat-screen games (Vatsal et al., 2024). This is due to VR technology’s increased sense of immersion and perception of reality. Being inside the gaming environment can trigger stronger emotional responses and more pronounced physiological changes (Lavoie et al., 2021; Lemmens et al., 2022).

The existing literature on virtual reality (VR) and mobile games (MO) is quite limited. Specifically, it remains unclear what physiological effects VR games that involve physical activity have on individuals (Peng et al., 2011; Brito-Gomes et al., 2014; Brito-Gomes et al., 2019). In addition, no study was found in the literature comparing VR games and MO games regarding physiological effects. In this context, it is important to know what kind of physiological load VR games that require body movement will bring compared to MO games that do not require movement. This study aimed to compare VR and MO games in terms of physiological load. We hypothesize that VR games will create a higher physiological load than MO games.

## 2 Material and methods

### 2.1 Research group

A total of twenty-two healthy university students (13 males and 9 females) participated in the study, with an average age of 21.72 ± 1.39 years, an average height of 171.59 ± 9.12 cm, and an average body weight of 64.95 ± 13.35 kg. The inclusion criteria for participants were: no smoking, no professional sports involvement, and no chronic diseases. There were no exclusion criteria related to participation in either of the two protocols. Prior to the study, all participants provided written informed consent.

### 2.2 Sample size

*A priori* power analysis was conducted using G*Power (version 3.1.9., Universität Düsseldorf, Germany) (Faul et al., 2007) to determine the required sample size for a one-tailed paired-samples t-test. The analysis was based on effect size of 0.8 (Cohen, 1988), an alpha level (α) of 0.05, and a desired statistical power (1 - β) of 0.95. The results indicated that a minimum sample size of 19 participants was required to achieve adequate power. Considering the loss of data, the study was conducted with 22 volunteers. Since all participants completed the study, there was no data loss.

### 2.3 Research procedure

**FIGURE 1:**
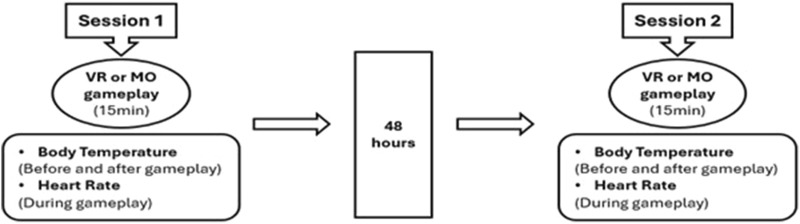
Data collection process.

The study used the crossover design method. Participants played an active video game (VR) using a virtual reality headset (Oculus Quest 2) and a mobile game (MO) on a smartphone on two separate days 48 h apart (Figure 1).

**FIGURE 2:**
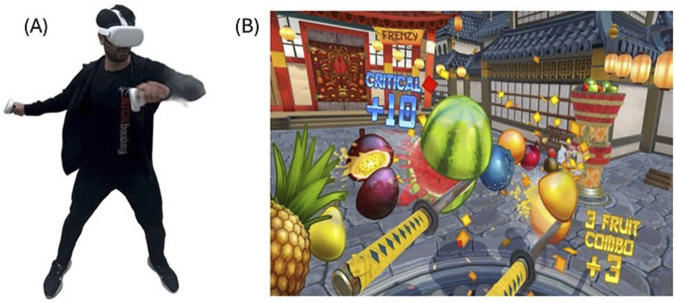
Experimental setup in an exercise setting and in-game active virtual reality (VR) scenes. **(A)** Playing with Oculus Quest 2 VR Headset, **(B)** Gameplay during Fruit Ninja 2.

In both game methods, “Fruit Ninja 2” (Halfbrick Studios. Brisbane, AUS) was selected for the game. Fruit Ninja 2 was chosen because it is popular among virtual reality (VR) games and provides users with a higher level of interaction and engagement (Coutu et al., 2020), as well as being a game that is equivalent to exercising on the elliptical and corresponds to moderate to vigorous exercise intensities (Stewart et al., 2022) that meet health benefit guidelines. The VR game was played in an empty room. The playground is designated with strips and necessary safety precautions have been taken. In this game, players hold a virtual sword in each hand and try to slice the fruits that appear in the air. The player tries to slice as many fruits as possible within the given time limit. The slicing is done by holding the game console apparatus in the VR game (Figures 2A, B), whereas in the MO game, it is done with finger movements. Participants also played MO games standing up. When the literature was examined, it was seen that the Fruit Ninja game was usually played for 10–15 min (Stewart et al., 2022; Coutu et al., 2020; Aygün and Çakır-Atabek, 2023; Yoo et al., 2017) and from this point of view, the game duration was chosen as 15 min to better understand the physiological load of the game in this study. Participants were randomly assigned which game to play first. The games were numbered and written on paper, and the papers were folded and placed in a box. Participants randomly determined the order of the game they would play by drawing numbers from the box before the game.

Heart rate (HR) and body temperature measurements were taken for participants before and after both games. Participants were instructed not to engage in high-intensity exercise for at least 2 days prior to the study and to avoid any stimulants, such as alcohol and caffeine, before the protocols. Ethical approval was obtained from the committee to ensure that the experimental procedures were appropriate.

### 2.4 Body temperature (BT)

A non-contact infrared thermometer (Zhao and Bergmann, 2023), which is widely used in the healthcare sector due to its accuracy, reliability, practicality, and ability to provide rapid results, was preferred for measuring participants’ body temperature. Accordingly, body temperature was measured from the forehead using a Saubern BNT9603 (Saubern, China) non-contact infrared thermometer according to the manufacturer’s instructions. The data obtained were recorded at a temperature of Celsius.

### 2.5 Heart rate (HR)

Heart rate (HR) data were recorded at RR (beat-to-beat) intervals with a Polar RS800CX telemetric device (Polar Electro Ov, Kempele, Finland). The device consists of a telemetric band worn on the chest in contact with the skin, a transmitter attached to it, and a watch worn on the arm. Polar monitors have been validated as a valid device for heart rate measurement (Vasconcellos et al., 2015; Barbosa et al., 2016). During the 15-min game, the heart rate data obtained from the participants was analyzed using the Polar Pro Trainer 5 software included with the device. The data obtained from the software included HR_max_ (the highest HR reached during the game), HR_mean_ (the average HR during the game), and HR_total_ (the total number of HRs reached during the total game), as well as the time spent 50% and below [under very light (UVL)], 50%–60% [very light (VL)], and 60% and above [above very light (AVL)] of the maximal HR determined according to the 220-age formula (Polar RS800CX User Manual; (Bushman and Bushman içinde, 2017)).

### 2.6 Statistical analysis

Statistical analyses were performed in IBM SPSS for Windows (v23, Armonk, NY, United States). Means, standard deviations, mean rank, and sum of rank values were used in the data presentation. The Shapiro-Wilk test, known for its high statistical power and sensitivity, was chosen to assess normality (Razali and Wah, 2011). According to the normality results, the Paired Samples T-test was used for comparisons of body temperature, HR_max_, and VL for VR and MO data, and the Wilcoxon Signed Ranks Test, which is the nonparametric equivalent, was used for comparisons of other data (HR_mean_, HR_total_, UVL, and AVL). Shapiro-Wilk significant (p) test results were body temperature (0.245), HR_max_ (0.500), and VL (0.067), while HR_mean_ (0.013), HR_total_ (0.013), UVL (0.030), and AVL (0.02). The Cohen’s d, and r (r = z/ √n ) were used to determine the effect sizes. Statistical significance was accepted as 0.05 in all the analyses.

## 3 Results

The participants’ body temperature values did not change before and after the game. Similarly, when VR and MO games were compared in terms of body temperature (Table 1), no difference was found before and after the game (p > 0.05).

**TABLE 1:** Body Temperature (^°^C) comparison before and after VR and MO games.

	Pre-game		Post-game		Test statistics	
Game	M	SD	M	SD	t	*p*
VR	36.63	0.11	36.63	0.12	0.001	1.000
MO	36.64	0.07	36.65	0.11	−0.530	0.602
	t = −0.513		t = −0.901			
	p = 0.613		p = 0.378			

VR: virtual reality; MO: mobile; M: mean; SD: standard deviation.

**FIGURE 3:**
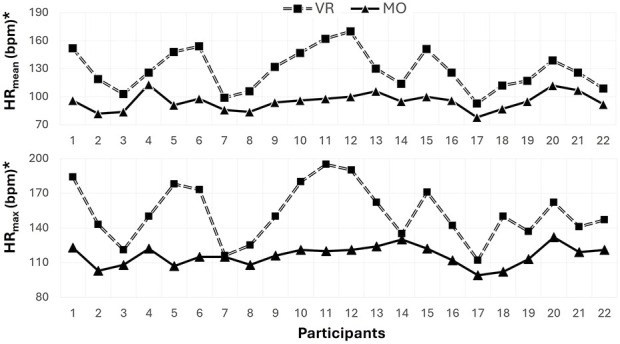
Comparison of VR and MO games regarding HR_mean_ and HR_max_.

Figure 3 presents an analysis of the average heart rate during the 15-min game, revealing that the heart rate in the VR game was significantly higher than in the MO game (z = −4.109, p < 0.01, r = 0.62). Similarly, participants reached higher maximal heart rates while playing the VR game, indicating a greater physiological load (t = 7.934, p < 0.01, Cohen’s d = 2.03) (Figure 3). Participants completed the VR game with a total heart rate of 1934.55 ± 323.53 heartbeats, whereas they completed the MO game with 1424.27 ± 140.23 heartbeats (Table 2). Statistical analysis showed that the total heart rate during the VR game was significantly higher with a large effect size (z = −4.107, p < 0.01, r = 0.62).

**TABLE 2:** Comparison of VR and MO games regarding physiological load.

Variables	Game	M ± SD	Mean rank N/P/T	Sum of rank N/P/T	Test statistics	*p*	*r/Cohen’s d*
HR_mean_ (BPM)	VR	128.86 ± 21.59	11.50/0.00/—	253.00/0.00/—	z = −4.109	0.001*	0.62
	MO	95.00 ± 9.31					
HR_max_ (BPM)	VR	152.91 ± 24.10			t = 7.934	0.001*	2.03
	MO	116.05 ± 8.80					
HR_total_	VR	1934.55 ± 323.53	11.50/0.00/—	253.00/0.00/—	z = −4.107	0.001*	0.62
	MO	1424.27 ± 140.23					
Under Very Light (<%50) (mm:ss)	VR	01:40 ± 03:06	1.00/11.50/—	1.00/230.00/—	z = −3.980	0.001*	0.60
	MO	09:24 ± 05:07					
Very Light (%50–60) (mm:ss)	VR	03:45 ± 03:44			t = −1.055	0.304	
	MO	05:29 ± 05:00					
Above Very Light (>%60) (mm:ss)	VR	09:34 ± 05:39	10.50/0.00/—	210.00/0.00/—	z = −3.920	0.001*	0.59
	MO	00:05 ± 00:11					

* p < 0.05, M: mean; SD: standard deviation; N/P/T: negative/positive/ties; BPM: beats-per-minute; mm:ss: minute:second.

**FIGURE 4:**
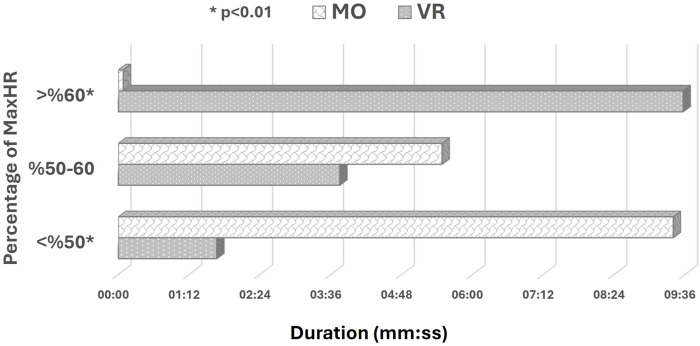
VR-MO comparison according to time spent in percent maximal heart rate.

Analyses according to the time spent in the percentage of maximal heart rate showed that the time spent at UVL (<50% of maximum heart rate) intensity was significantly longer in the MO game compared to the VR game (z = −3.980, p = 0.001, r = 0.60). In contrast, the time spent at AVL (>60% of maximum heart rate) intensity was significantly longer with a large effect size in the VR game compared to the MO game (Figure 4) (z = −3.920, p = 0.001, r = 0.59). In contrast, the two games had no significant difference regarding duration at VL (50%–60% of maximum heart rate) intensity (t = −1.055, p = 0.304). These findings indicate that VR games have generated a significantly higher physiological load on participants with a larger effect size at AVL intensity.

One of the key findings of this study was related to participant game preferences. After playing both VR and MO games, participants were verbally asked, “Which type of game would you prefer to play more?” Their answers were recorded, and 21 out of the 22 participants indicated that they preferred VR games.

## 4 Discussion

As digital gaming technologies advance rapidly, it is essential to explore the physiological impact of virtual reality (VR) games that involve physical activity compared to mobile games that do not require movement. This topic is significant for both e-sports research and health and exercise science. By comparing the physiological effects of VR and MO games, we can better understand how physical activity in a virtual environment influences individuals.

This study aimed to compare the effects of VR and MO games on physiological load. The findings indicate that players experienced higher heart rates while playing VR games than MO games. Measured parameters such as HR_mean_, HR_max_ reached during the game, and HR_total_ were higher in VR games. Although the time spent at AVL (>60% of maximum heart rate) intensity was longer in VR games and more intense regarding physical activity, 21 participants preferred VR games.

There were no significant differences in body temperature results, either within the groups or between them. This lack of difference was expected in MO games. Currently, there is no direct data on the impact of MO games on body temperature. However, since MO games typically do not involve as much physical activity as virtual reality (VR) games, they are not likely to affect body temperature as pronounced. Although participants may be standing in MO games, the only physical movements are limited to their fingers and wrists, resulting in minimal overall exertion. The involvement of different muscle groups during exercise can lead to differences in the body’s thermoregulation mechanisms. This variation stems from the distinct efferent nerve stimuli that reach the thermoregulation center when performing exercises with the arms versus the legs. In the VR game mentioned in the protocol, participants engaged with the Fruit Ninja 2 game by holding the console and using their arms. Given the physical activity required in VR games, it is noteworthy that there was no significant difference in body temperature compared to traditional MO games. However, even at an equivalent level of energy expenditure and heat production, body temperature increases during arm exercises were lower than those observed during leg exercises (Asmussen and Nielsen, 1947). Conversely, another study indicated that arm exercises elicited higher heart rates and greater perceived exertion than leg exercises (Pivarnik et al., 1988). The existing literature suggests that while VR games may not directly influence body temperature, they can impact perceived temperature (Lauderdale, 2017; Morgan et al., 2024).

The effects of VR games on heart rate can vary based on the type of game and how it is played. Generally, VR games can increase heart rate (Naugle et al., 2024), influenced by both the game’s intensity and the player’s experience. For example, Godfrey et al. (2024) and Evans et al. (2021) reported that the game Beat Saber provides light to moderate-intensity exercise, while Thrill of the Fight offers an intensity comparable to high-intensity workouts. Immersive games, particularly in virtual reality (VR), can significantly increase the physiological demands on players. VR games enhance the player’s sense of presence (Coxon et al., 2016; Lin, 2017), making them feel more detached from reality and deeply engaged in the virtual environment. Research indicates that VR games elicit stronger feelings of presence than traditional TV screens (Wirth et al., 2007; Cummings and Bailenson, 2016). In their literature review, Sousa et al. (2022) noted that immersive VR games generally induce activity levels that are classified as moderate to high intensity.

VR games provide a more vivid and dynamic experience than MO games, which might explain why participants exert more effort during these activities. Liu et al. (2024) conducted a study involving university students, where they tested three different biking protocols: immersive VR cycling, non-immersive VR biking, and traditional stationary biking. The results indicated that VR cycling resulted in significantly higher vitality levels than screen and traditional cycling (Liu et al., 2024). Similarly, Zeng et al. (2022) compared VR-assisted exercise, exergaming, and traditional cycling regarding perceived difficulty and the number of pedal cycles performed by university students. Their findings revealed that participants perceived VR-assisted exercise cycling as less challenging than the other methods. However, they completed more pedal cycles than exergaming and traditional cycling (Zeng et al., 2022).

The game used in this study, Fruit Ninja 2, is a virtual reality (VR) game where participants attempt to slice fruits thrown into the air using the controls of a gaming console. The dynamic nature of the game encourages participants to use their hands and arms intensively while also stepping and turning their bodies in various directions to enhance their physical mobility. This indicates that VR games necessitate more physical activity and intense muscle engagement compared to traditional seated MO games. One study assessed the physical activity intensity of healthy adults playing a VR game that allows for locomotor movements and the Icaros flight simulator using a 1-7 Likert scale. The researchers found that the exercise intensity in the VR game was higher (Dębska et al., 2019). Additionally, another study involving obese children compared a game requiring locomotor movement to a VR game that limited movement. The results indicated that the heart rates were significantly higher during the game requiring locomotor movement (Polechoński et al., 2020).

Although we did not use a pedometer to measure activity levels in this study, we observed that participants moved significantly more while playing the VR games. It was found that VR-based games not only increased the physical mobility of the players but also significantly elevated the intensity of their physical activity during the game. Consequently, we can conclude that participants experienced higher intensity levels while engaging in VR games. The findings from our study, which include elevated HR_mean_, HR_max_, HR_total_, and a significantly longer time spent at AVL intensity, align with existing literature (Polechoński, 2024; Polechoński et al., 2022).

Another key finding regarding exercise intensity in this study is the time spent in maximal heart rate percentiles. At UVL intensity (<50% of Maximum Heart Rate), participants spent significantly more time in the MO game compared to the VR game. Similarly, although no statistically significant difference was found, the time spent at VL intensity was also longer in the MO game than in the VR game. This indicates that in the MO game, participants tended to play with minimal movement and often stopped, resulting in less physical effort. Consequently, this supports the idea that participants experience a lower physiological load in MO games compared to VR games. In contrast, the time participants spent in the VR game at AVL intensity (>60% of Maximum Heart Rate) was significantly longer than in the MO game (Figure 4). This level of intensity can be considered moderate or higher. One study reported that table tennis training in VR arcade mode corresponded to a moderate level of physical activity, averaging 69.50% of HR_max_. Furthermore, Polechoński et al. (2022) investigated how adding weight to participants’ arms during active VR games affected physical activity intensity. They had the participants play the game with and without 0.5 kg on their arms. Their results showed that playing the game without added weight corresponded to 63.7% of HR_max_ (Polechoński et al., 2022). These findings suggest that VR gaming creates a more significant physiological load on participants at very light and higher intensity levels, and our findings are consistent with the literature (Stewart et al., 2022; Polechoński, 2024; Polechoński et al., 2022).

## 5 Conclusion

This study aimed to compare the effects of VR games and MO games on physiological load. The results indicate that VR games require greater cardiovascular effort and result in a more significant physiological load on players than MO games. Specifically, parameters such as HR_mean_, HR_max_ reached during the game, and HR_total_ were higher in VR games. Additionally, the time spent at AVL intensity was significantly longer in VR games than in MO games. Consequently, VR games created a higher physiological load than MO games. The heart rate parameters measured during VR games were elevated across all levels, suggesting that VR may be an effective tool for promoting physical activity through gaming experiences.

These results suggest that VR games have significant potential to increase physical activity levels and to be used as a tool in exercise-related interventions. However, it is important to consider the specific differences between various game types and choose games suitable for individuals’ physical abilities. Additionally, long-term use of VR headsets in children under a certain age may negatively impact postural stability and increase the symptoms of motion sickness, so monitoring the duration of gameplay is essential. Since VR games can significantly affect children’s psychological safety, educators and parents should carefully evaluate the content of these games. Lastly, creating a large, obstacle-free play area is recommended to minimize the risk of bumps or falls.

## 6 Future directions and limitations

A more comprehensive investigation into the physiological effects of VR and MO games will be a crucial focus for future studies. Additionally, long-term follow-up research could explore how both VR and MO games influence physical fitness, cardiovascular health, and overall physical activity levels over time. Beyond the physiological impact, it is also important to examine how these games affect players’ motivation, engagement, and enjoyment. By understanding these factors, we can use games more effectively in health and exercise programs. The study assessed the physiological effects of gaming during 15-min sessions. However, a more extended follow-up period is necessary to better understand the impacts of long-term gaming habits. Additionally, the evaluation focused only on certain types of VR and MO games. Variations in game types and mechanics can significantly influence their physiological effects. Therefore, future research could explore a wider range of game types. Moreover, the study used limited measures, focusing solely on basic physiological parameters like heart rate. Other important factors, such as energy expenditure, oxygen consumption, lactate levels, and perceived exertion, were not evaluated. Acknowledging these limitations will enhance the interpretation of the current findings and improve the design of future studies.

## Data Availability

The raw data supporting the conclusions of this article will be made available by the authors, without undue reservation.
